# Very Rare Liver Neoplasm: Lymphoepithelioma-Like (*LEL*) Hepatocellular Carcinoma

**DOI:** 10.1155/2018/2651716

**Published:** 2018-09-06

**Authors:** Marcello Filotico, Valentina Moretti, Federica Floccari, Alessandro D'Amuri

**Affiliations:** ^1^Anatomic Pathology Unit, “Card. G. Panico” Hospital, Tricase (LE), Italy; ^2^Clinic Pathology Unit, “Camillo De Lellis” Hospital, Manfredonia (FG), Italy

## Abstract

A case of lymphoepithelioma-like (LEL) hepatobiliary carcinoma is reported. To date, only 89 cases of this rare neoplasm have been reported, with both hepatocellular and cholangiocellular histotype. The case reported here could be classified as LEL mixed hepatobiliary carcinoma (*Hepato-Cholangio*), a histotype not reported so far in the LEL variant.

## 1. Introduction

The lymphoepithelioma-like hepatocellular carcinoma (*or inflammatory carcinoma*) is a rare primary neoplasm of the liver, usually poorly differentiated, in which the neoplastic component is densely infiltrated by lymphoid cells.

## 2. Case Report

A 62-year-old woman with HBV-related hepatopathy has been suffering for a few months of pain and a sense of weight in the right hypochondriac site. A CT scan reveals a lesion of about 5 cm between the VI hepatic segment and right colon. Colonoscopy demonstrates near the right colonic flexure an ab* extrinseco *compression that dislocates the bowel and makes the endoscope's progression difficult. The most significant laboratory tests were AFP = 394.90 ng / ml (*normal value <15*); CEA = 2.20 ng / ml (*normal value <5*). Antibodies to HBV were positive. An exophytic, encapsulated neoformation with a diameter of 5 cm, at the level of the VI hepatic segment, is found in the laparoscopic procedure. The segment is resected by ultrasound scalpels. Without complications, the patient is discharged the day after the operation.

## 3. Materials and Methods

Numerous fragments are taken from the surgical sample, fixed in formalin, and embedded in paraffin. The sections are stained with H&E and subjected to silver impregnation according to Gomori. A large panel of antibodies is used for immunohistochemistry.

### 3.1. Macro

**Figure 1:**
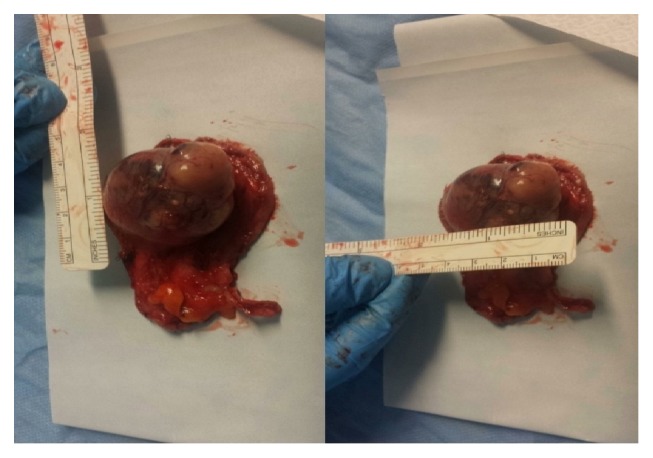
Surgical specimen: grossly polypoid neoformation with a large implant base.

The surgical sample consists of a globular neoplasm, polypoid in shape with a diameter of about 5 cm., and a large peduncle attached on a triangular flap of hepatic parenchyma (Figure 1). The neoformation has a smooth and shiny surface, of a reddish-brownish color. The cut surface is reddish-brownish, opaque grainy, intersected by coarse fibrous bands.

### 3.2. Histology

**Figure 2:**
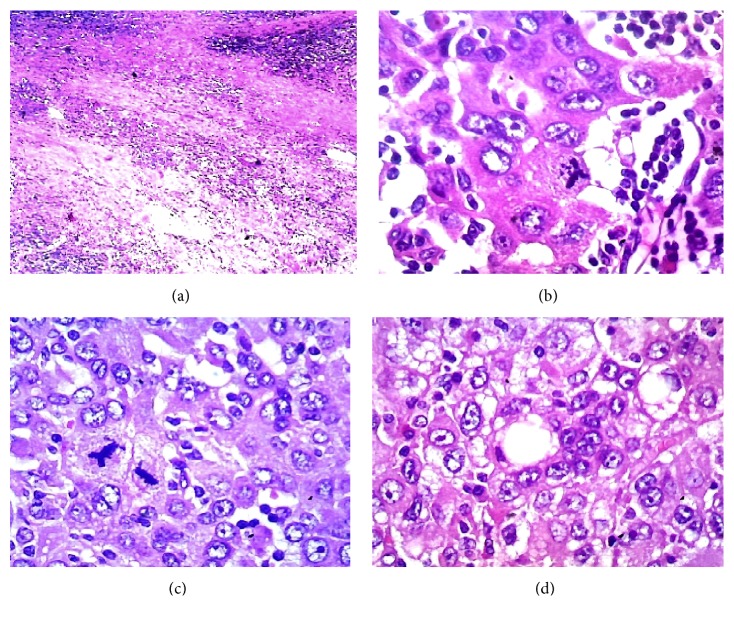
(a) Fibrous band delimiting nodular area (*HE 40x*); (b) globular-polyhedral epithelial elements, with a pink, foliaceus, cytoplasm (*HE 200x*); (c) high mitotic activity, atypical mitotic figures (*HE 200x*); (d) trabecular arrangement with acinar formation (*HE 200x*).

**Figure 3:**
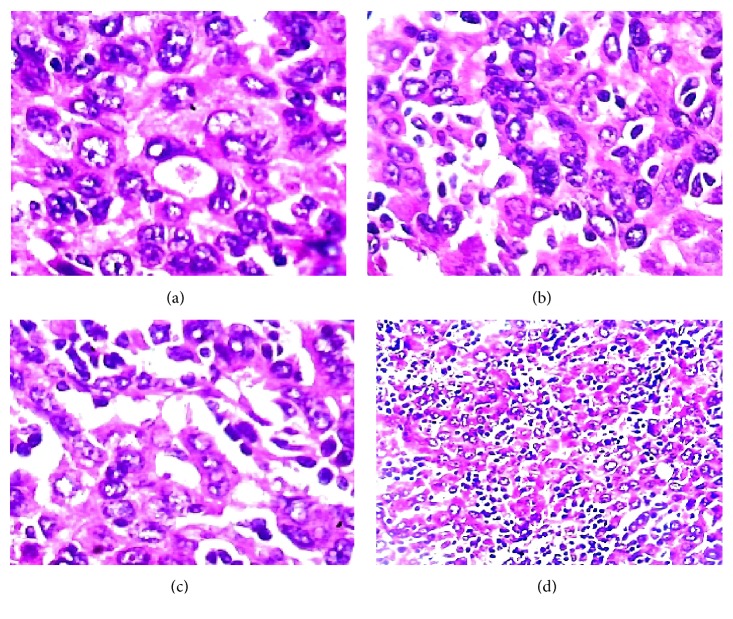
(a, b, c) Abortive tubular formations (*HE 200x*); (d) massive lymphoid infiltration occupying sinusoidal space and disrupting the trabecular architecture (*HE 100x*).

**Figure 4:**
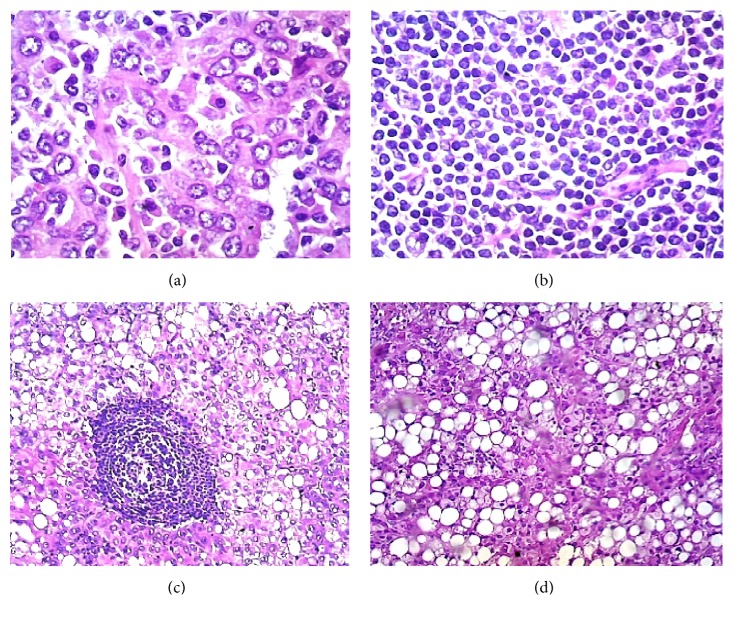
(a) Lymphoid cells invading sinusoidal spaces (*HE 200x*); (b) diffuse lymphoid infiltration (*HE 200x*); (c) nodular lymphoid aggregate (*HE 200x*); (d) area of steatosis (*HE 100x*).

**Figure 5:**
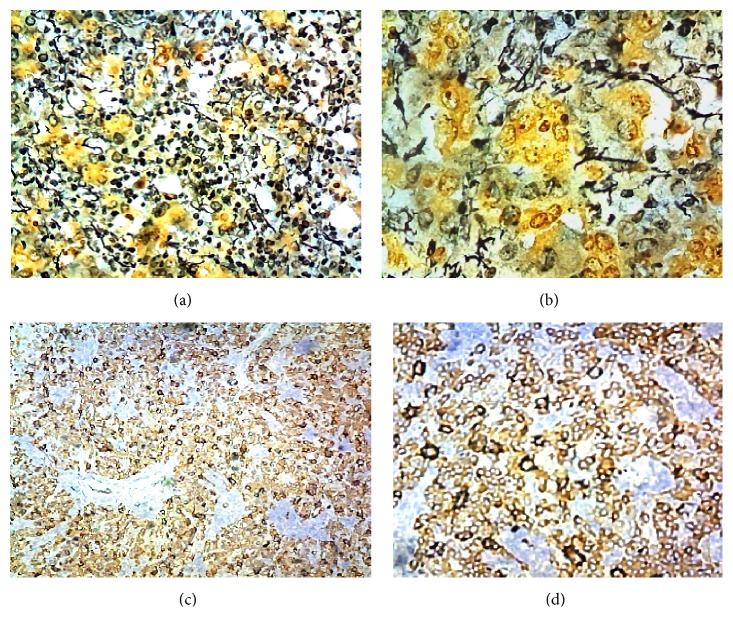
(a) Argyrophilic reticular network interrupted (*Gomori 100x*); (b) Gomori (*400x*); (c-d) Cytokeratin AE1-AE3—it is recognizable, though the trabecular architecture is altered ((c)* 100x, *(d)* 200x*).

**Figure 6:**
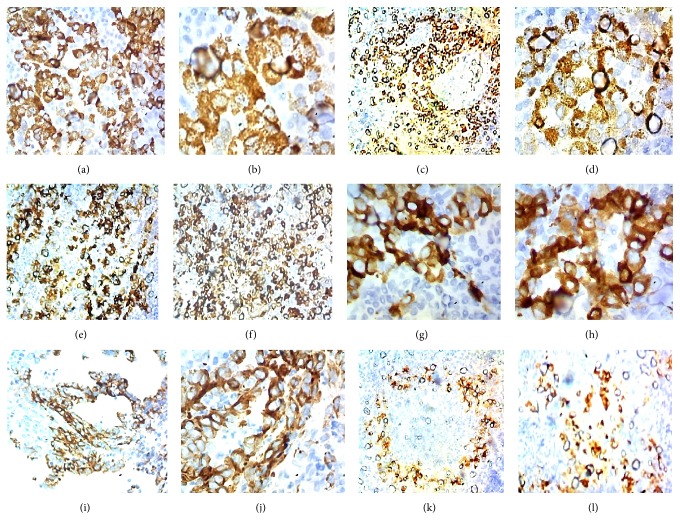
(a-b) Hepatocyte diffuse and intense positivity (*100x,200x*); (c-d) TTF1, focal positivity (*100x, 200x*); (e-f) CK7 focal positivity (*100x*); (g-h) CK7 positivity in ductular structures (*400x*); (i-j) CK19 positivity in canalicular structures (*100x, 200x*); (k-l) *α*FP focal positivity (*100x*).

**Figure 7:**
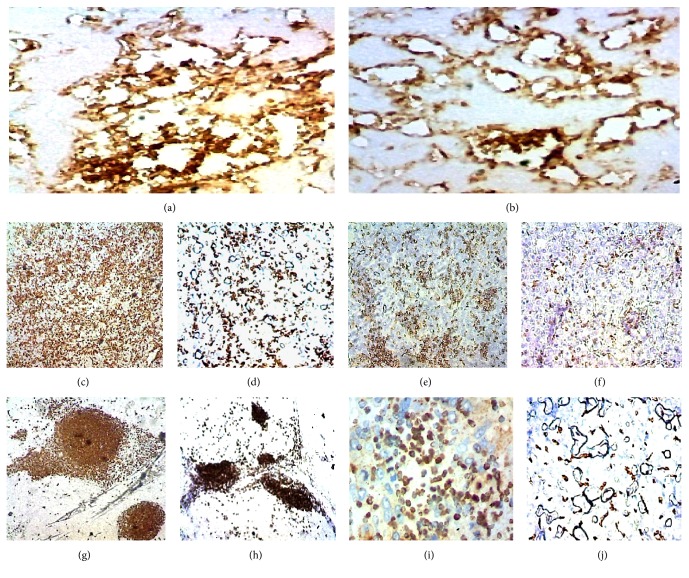
(a-b) CD10;(*200x*); (c) CD3; (d) CD4; (e) CD5; (f) CD8; (g-h) CD20; (i) CD138; (j) CD34 (*100x*).

The normal liver structure is no longer recognizable. Just below Glisson's capsule are ductular biliary structures. The tissue is crossed by wide bands of fibrous tissue surrounding grossly nodular areas (Figure 2(a)). The bulk is made up of disorderly proliferation of unequivocally neoplastic roundish, polyhedral epithelial elements, with a pink, foliaceus, cytoplasm (Figure 2(b)). The nucleus is voluminous and hyperchromatic, and nucleoli are frequently seen. There is high mitotic activity including atypical mitoses (Figure 2(c)). Occasionally in some areas a vaguely trabecular structure is still recognizable along with a few abortive tubular formations (Figures 2(c), 3(a), 3(b), and 3(c)) These cells are dissociated by a massive lymphoid infiltrate consisting of small and medium size, mononuclear cells, sometimes with plasmacytoid appearance, which occupy the sinusoidal spaces (Figures 3(d) and 4(a)), up to render scarcely visible the epithelial component (Figure 4(b)). Within close to fibrous bands, the lymphoid infiltrates aggregate into nodular formations (Figure 4(c)). There are also large areas of steatosis and coagulative necrosis (Figure 4(d)). The argyrophilic network is fragmented (Figure 5(a)). As a consequence of this phenomenon, the epithelial component is fragmented into a small group of few cells or even isolated cells (Figure 5(b)). The immunohistochemical investigations demonstrate the existence of two epithelial cell populations: one, the majority, expressing CKs AE1/AE3 (Figures 5(c) and 5(d)), Hepatocyte, (Figures 6(a) and 6(b)), TTF1 (Figures 6(c) and 6(d)), AFP (Figures 6(k) and 6(l)), CD10 (Figures 7(a) and 7(b)), and another, minor cell population expressing CK7 (Figures 6(e), 6(f), 6(g), and 6(h)) and CK19 (Figures 6(i) and 6(j)). The diffusely present lymphoid component mainly contains elements with phenotype T: CD3 (Figure 7(c)), CD4 (Figure 7(d)), CD5 (Figure 7(e)), and CD8 (Figure 7(f)), while the phenotype B: CD20 is limited to the nodular aggregates in the fibrous bands (Figures 7(g) and 7(h)) and CD138 to the plasmocytoid elements (Figure 7(i)). CD 34 highlights irregular sinusoids (Figure 7(j)). The morphological and immunohistochemical data allow placing the lesion in the category of the so-called ***lymphoepithelioma-like hepatocellular carcinoma.***

## 4. Discussion and Conclusions

The lymphoepithelioma-like (*LEL*) hepatocellular carcinoma (HCC) (*or inflammatory HCC*) have only recently been recognized as an entity in itself [1]. The first report dates back to 1995 [2]. The definition of lymphoepithelioma-like HCC has been present in the literature since 1996 [3]. To date, 86 cases have been reported, in 59 of which the epithelial component shows the morphological characteristics and the immunophenotypic profile of* HCC *and in 27 of cholangiocellular carcinoma (*CC*) [4]. On the basis of similar lesions occurring in various sites (*lung, urinary bladder, vagina, uterus, skin, etc.*), all referring to the basic model in nasopharyngeal localization, from the earliest observations has sought and found an association with EBV [5]. Between LEL-HCC and LEL-CC significant differences have been reported. The male sex prevails in the LEL-HCC (*64%*), while the female prevails in the LEL-CC (*66%*). The Caucasians prevail in LEL-HCC (*65%*), while Asians prevail in the LEL-CC (*92%*). EBV is reported in 2% of cases of LEL-HCC and in 74% of cases of LEL-CC. Cirrhosis is present in 46% of cases of LEL-HCC and in 19% of cases of LEL-CC. Positivity for HBV is present in 30 and 40% of cases, respectively, while HCV is 34% and 7% [6]. In summary the most frequent profile of a patient with LEL-HCC is that of a Caucasian male, carrying an HBV or HCV infection with cirrhosis, while that of a patient with LEL-CC is of an Asian female carrier of an EBV infection, rarely associated with cirrhosis. Since the first observations, several reports have been dedicated to investigating the meaning of the lymphoid infiltrate. The assumption that this phenomenon represents a defensive process against the neoplasm could explain the less unfavorable prognosis of this type of lesions. In literature, the lymphoid infiltrate is described as widespread in parenchymal areas and in nodular aggregates in the context of fibrous bands. Immunohistochemically, most of the infiltrating lymphocytes, other than those in the lymph follicle, were identified as T lymphocyte with a prevalence of CD4 and CD8. The better prognosis of the LEL-HCC group could attribute to the antitumor effect induced by the cellular immunity of CD8 + and CD4 + T lymphocytes, and partly by the humoral immunity of B cells which formed lymph follicles [7]. In a report of 8 LEL-HCC cases, free from viral infections of any kind, the lymphoid infiltrate is made up mainly of T cells CD4 +, CD8 +: This data would rule out an intervention of the viral genome in determining the immune response which would, therefore, be determined by the tumor. In the same note, it is emphasized that the immunohistochemical investigation in three of these cases demonstrates a focal or diffuse expression of CK19, the characteristic of cholangiolar differentiation. Strangely, the evaluation of the epithelial component is limited to CK19 without the use of HCC-specific markers (*Hepatocyte, Hep Par 1, CD10*), which may have been allowed to label those cases as CC or as mixed forms [8]. From some authors, the phenomenon is interpreted as an attempt to spontaneous regression of the tumor. In support of this hypothesis, a disease-free patient was reported 60 months after tumor resection [9]. The immunophenotypic evaluation of our case is substantially consistent with the literature, with the exception of a lower number of CD8 + lymphocytes. From the literature it is clear that the phenomenon of lymphoid infiltration can affect both types of hepatobiliary neoplasms following with their epidemiological distribution. As well it is evident that, in the majority of cases, they were poorly differentiated neoplasms. The immunophenotypic evaluation of the epithelial component of our case highlights the expression of antigens related to HCC and also antigens related to CC. The poorly differentiated morphology was further altered by the massive lymphoid infiltration, which did not help in identifying two distinct components. The expression of specific hepatocellular antigens (*Hepatocyte, CD10, αFP*) is sufficient to affirm the presence of a hepatocellular differentiation, just as it cannot be said for CC because CK7 and CK19 do not explicitly prove a biliary differentiation of a proliferation as these antigens can also be expressed by the hepatocellular component [10]. It should be stressed, however, that the lumen of the rare and abortive tubular structures present is delimited by smaller cuboidal cells expressing antigens CK7 (Figures 6(g) and 6(h)) and CK19 (Figures 6(i) and 6(j)).

This finding would favor the possibility of the existence of a true cholangiocellular component. Liver tumors of mixed hepatocellular and biliary phenotype were, until recently, referred to as* combined hepatocellular and cholangiocarcinoma (mixed hepatocellular-cholangiocarcinoma)*. Under these terms were classified both “*collision*” tumors, which coexist in the same organ, two distinct tumors hepato- and cholangiocarcinoma, both single tumors with mixed morphologic features of both hepatocellular and biliary differentiation. The International Consensus Panel advises reserving the denomination of* Mixed Hepatobiliary Tumors* only to single tumors with mixed morphologic features, distinguishing in it two subgroups according to the presence or absence of stem cells [11]. Tumors that carry stem cells* (ckit +) *are, in turn, depending on the morphology classified as intermediate, transitional, and small cell [12]. No case of LEL mixed hepatobiliary carcinoma (*hepato-cholangiocellular carcinoma*) according to the previous definition hitherto has been reported in the literature. A single report of mixed LEL-carcinoma describes a case in which a well-differentiated cholangiocellular component coexisted with one second poorly differentiated. Immunohistochemical studies revealed that both components were immune reactive for CKAE1/AE3, cytokeratin 7, cytokeratin 19, and, focally, monoclonal CEA. Both components were negative for cytokeratin 20 and HePar1. This lesion according to the aforementioned criteria cannot be considered to fall within* Mixed Hepatobiliary Tumors *[13]. The case of our observation, however, can rightfully be classified as a ***LEL Mixed Hepatobiliary Tumor without stem cells***, as immunophenotypically the two components are well represented and in some areas a trabecular architecture (Figures 2(b) and 2(c)) and some tubular formation (Figures 3(b) and 3(c)), CK7 (Figures 6(g) and 6(h)) and CK19 + (Figures 6(i) and 6(j)), can be identified. To the best of our knowledge, this would be the first case of LEL Mixed Hepatobiliary Tumor reported in the literatures.
